# Efficacy and safety of exercise for hypertrophic cardiomyopathy: a systematic review and meta-analysis

**DOI:** 10.1080/08998280.2026.2667666

**Published:** 2026-05-18

**Authors:** Ahmed Mazen Amin, Wafaa Shehada, Mohamed Elgebaly, Dina Ayman, Mahmoud Mahmoud Ibrahim, Basel Abdelazeem, Kevin Felpel

**Affiliations:** a Faculty of Medicine, Mansoura University, Mansoura, Egypt; b Faculty of Medicine, Islamic University of Gaza, Gaza, Palestine; c Faculty of Medicine, Beni-Suef University, Beni-Suef, Egypt; d McLaren Health Care, Flint, Michigan, USA; e Michigan State University, East Lansing, Michigan, USA; f Department of Cardiology, West Virginia University, Morgantown, West Virginia, USA

**Keywords:** Cardiorespiratory fitness, exercise, hypertrophic cardiomyopathy, oxygen consumption, patient safety, quality of life

## Abstract

**Background:**

Patients with hypertrophic cardiomyopathy (HCM) are generally restricted from exercising. We aimed to investigate the effects of exercise on cardiorespiratory fitness, cardiac structure, cardiac function, and clinical safety for patients with HCM.

**Methods:**

We performed a systematic review and meta-analysis by searching PubMed, Web of Science, Scopus, Embase, and CENTRAL up to April 2025. Dichotomous outcomes were pooled using risk ratios (RRs), while continuous outcomes were pooled using mean differences (MDs) or standardized mean differences (SMDs), all with 95% confidence intervals (CIs), using R version 4.3.1.

**Results:**

With the inclusion of seven studies—three randomized controlled trials and four observational studies—our cohort comprised 9702 patients. Compared to the control group, exercise was significantly associated with increased cardiorespiratory fitness (peak oxygen consumption [peak VO_2_] (mL∙kg^−1^∙min^−1^) (MD: 1.78; 95% CI [0.98, 2.59]; *P* < 0.01) and VO_2_ at an anaerobic threshold (mL∙kg^−1^∙min^−1^) (MD: 1.27; 95% CI [0.34, 2.20]; *P* < 0.01), reduced body mass index (kg/m^2^) (MD: −0.67; 95% CI [−1.17, −0.18]; *P* < 0.01), reduced left ventricular wall thickness (mm) measured by echocardiography (MD: −0.56; 95% CI [−1.01, −0.11]; *P* = 0.01), increased quality of life physical factor (SMD: 0.66; 95% CI [0.34, 0.97]; *P* < 0.01), and decreased incidence of all-cause mortality (RR: 0.71; 95% CI [0.60, 0.85]; *P* < 0.01).

**Conclusion:**

This study suggests that structured exercise interventions are safe and associated with improvements in cardiorespiratory fitness, left ventricular wall thickness, body mass index, and quality of life physical factor in patients with HCM, without increasing the risk of arrhythmias. Although a lower mortality rate was observed, this finding was mainly driven by observational studies and should therefore be interpreted with caution. Larger randomized controlled trials are needed to confirm these findings.

Hypertrophic cardiomyopathy (HCM) is the most common inherited cardiovascular disorder, with an estimated prevalence between 1 in 200 and 1 in 500 individuals.^1^^,^^2^ It is characterized by unexplained left ventricular hypertrophy not attributable to abnormal loading conditions such as hypertension or aortic stenosis.^2^ Although many patients remain asymptomatic, others experience exertional dyspnea, angina, palpitations, syncope, or progress to heart failure, atrial fibrillation, or sudden cardiac death (SCD).^3^^,^^4^ Historically, physical activity has been restricted in patients with HCM due to concerns that exercise might provoke ventricular arrhythmias and increase SCD risk.^5^^,^^6^ As a result, many individuals with HCM are insufficiently active, with studies reporting that only about 45% meet recommended physical activity levels.^7^ Sedentary behavior in this population is associated with higher obesity prevalence, reduced cardiorespiratory fitness (CRF), and diminished quality of life (QoL).^8^^,^^9^

However, a growing body of evidence has opposed these longstanding restrictions in recent years. Prospective observational studies and randomized clinical trials (RCTs) suggest that appropriately guided exercise training can be both safe and beneficial in selected patients with HCM.^7^^,^^10–12^ Supervised exercise programs have been shown to improve functional capacity, reduce left ventricular filling pressures, and enhance ventilatory efficiency without increasing arrhythmic risk or inducing adverse remodeling.^11^^,^^13^ Moreover, participation in regular physical activity may help attenuate comorbidities such as obesity, hypertension, and diabetes, which are prevalent in this population and contribute to worsened cardiovascular outcomes. Objective measures of exercise performance, such as cardiopulmonary exercise testing, have been shown to provide prognostic value in HCM, highlighting the importance of functional assessment in studies comparing exercise interventions with control groups.^14^

Contemporary international guidelines now support integrating structured exercise into HCM management, provided decisions are individualized and guided by shared decision-making, thorough risk assessment, and expert supervision. Current guidelines endorse low- to moderate-intensity physical activity for most patients, while discouraging high-intensity training in those at elevated risk.^15–17^ Despite this shift in clinical approach, a comprehensive synthesis of the impact of exercise interventions, regardless of intensity or duration, on clinical, functional, and structural outcomes in patients with HCM remains lacking. To address this gap, we conducted a systematic review and meta-analysis investigating the impact of exercise on patients with HCM. In this study, we focused on CRF, defined by peak oxygen consumption (peak VO_2_) and VO_2_ at the anaerobic threshold (AT), measured or estimated as applicable, as our primary outcome, reflecting functional capacity and long-term prognosis. Moreover, given the historical caution regarding exercise in HCM, we also investigated the safety profile, including arrhythmic events, implantable cardioverter defibrillator (ICD) therapies, syncope, and other adverse cardiovascular events.

## METHODS

This meta-analysis adhered to the PRISMA statement guidelines for systematic reviews and meta-analyses^18^ and the Cochrane Handbook for Systematic Reviews and Meta-Analysis.^19^ We registered this meta-analysis prospectively in the International Prospective Register of Systematic Reviews (PROSPERO) under ID CRD420251106349.

### Data sources and study selection

A comprehensive search was conducted across five electronic databases: PubMed, CENTRAL, Web of Science (WoS), SCOPUS, and EMBASE up to April 2025. The search strategy combined relevant keywords and medical subject headings terms related to exercise interventions and HCM. The details of the search strategy are outlined in *Supplemental Table 1*.

We included any study that met our PICO criteria. The population (P) consisted of patients of all ages with HCM. The intervention (I) was exercise (irrespective of intensity or duration). The control (C) was usual care, usual activity, or sedentary life. The primary outcome (O) was the CRF: mean change in peak oxygen uptake (peak VO_2_) and VO_2_ at AT, a widely accepted measure of CRF and a prognostic marker in HCM.^14^^,^^20^ Secondary outcomes included measures of cardiac structure and function and clinical safety: body mass index (BMI), left ventricular wall thickness (LVWT), post-exercise left ventricular outflow tract (LVOT) gradient, exercise time, the ratio of the peak early filling velocity (E) to the peak velocity of the mitral valve annulus (e’) (E/e′), the ratio of peak velocity blood flow from left ventricle relaxation in early diastole (the E wave) to peak velocity flow in late diastole caused by atrial contraction (the A wave) (E/A), N-terminal pro-B-type natriuretic peptide (NT-proBNP), and QoL, as well as safety outcomes, including arrhythmic events, ICD therapies, syncope, and other adverse cardiac events.

All identified records were imported into Covidence software for reference management, and duplicates were removed. Four reviewers independently screened titles and abstracts for relevance, followed by a full-text review of potentially eligible studies. Disagreements were resolved by consensus.

### Data extraction

Three reviewers independently extracted data from the included studies into a Microsoft Excel sheet. The Excel sheet encompassed study characteristics (study design, country, total number of the included participants, description of the exercise arm, control arm, main inclusion criteria, exclusion criteria, primary outcome, composite outcome definition, and the follow-up duration); baseline patient data (number of patients in the exercise and the control groups, sex, age, BMI, left ventricular ejection fraction [LVEF], New York Heart Association [NYHA] functional classification classes, peak VO_2_), comorbidities (hypertension, diabetes, atrial fibrillation, and stroke or transient ischemic attack), and medications (beta-blockers and calcium channel blockers). Any discrepancies were resolved through discussion.

### Risk of bias

Three reviewers independently assessed the risk of bias for all included studies. The Cochrane Risk of Bias tool (RoB 2.0)^21^ was applied to RCTs. The Risk of Bias in Non-randomized Studies of Interventions (ROBINS-I) tool^22^ was used for observational studies. The RoB 2.0 tool addressed five domains: randomization, deviation from intended exercise or control protocols, missing outcome data, outcomes measurement process, and selection of the reported results. ROBINS-I judged seven domains: bias arising from confounding variables, selecting the study participants, classifying the interventions, bias arising from the deviation from intended interventions, bias due to missing outcomes, bias in measurement of the outcomes, and bias in selecting the reported results. Any conflicts were resolved by consensus.

### Statistical analysis

R version 4.3.1 was used to carry out statistical analysis. For continuous outcomes, pooled estimates were expressed as mean differences (MDs) or standardized mean differences (SMDs) with corresponding 95% confidence intervals (CIs). Risk ratios (RRs) with 95% CIs were calculated for binary outcomes. When studies reported zero events in both arms for a given outcome, a continuity correction was applied to enable inclusion in the meta-analysis. We used the random-effects model when there was a significant heterogeneity (I^2^ > 50%) and the common-effect model when heterogeneity was less (I^2^ < 50%). To assess heterogeneity, we used the chi-square and I-square statistics, where the chi-square test assesses the presence of heterogeneity, and the I-square statistic assesses its degree. We considered an alpha level < 0.1 for the chi-square test to denote significant heterogeneity. Sensitivity analysis was performed in cases of significant heterogeneity, excluding individual studies to evaluate their impact on the pooled estimates and heterogeneity. We also conducted subgroup analyses to compare outcomes across different exercise intensities (moderate versus high-intensity protocols) and between RCTs and observational studies. *P* values of outcomes < 0.05 were used to detect statistical significance.

## RESULTS

**Figure 1.**
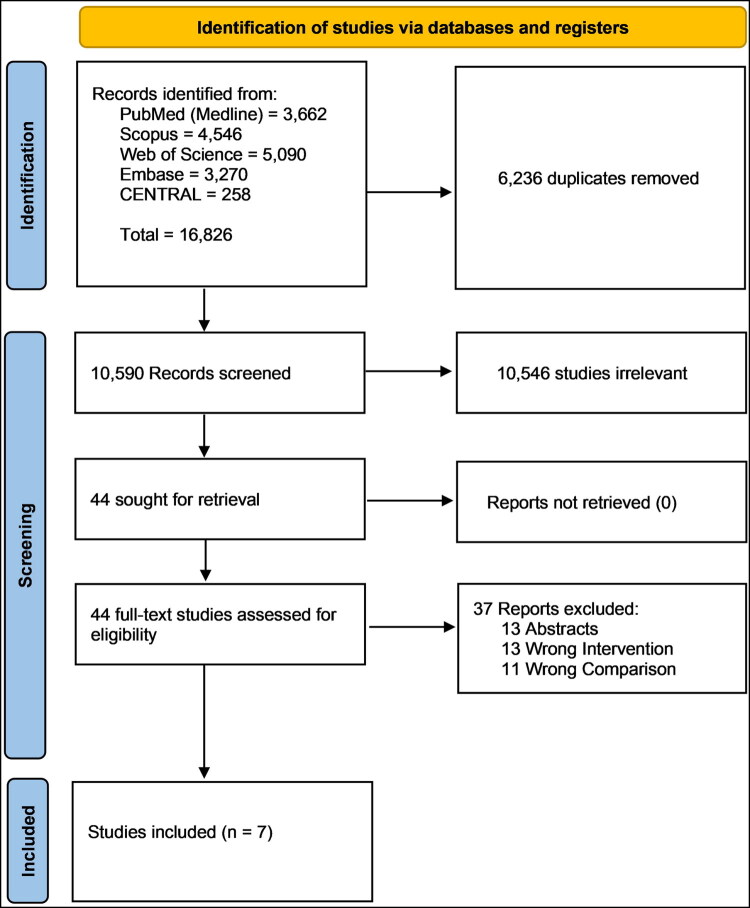
PRISMA flow chart of the screening process.

After searching PubMed, CENTRAL, Scopus, WoS, and EMBASE, the search retrieved 16,826 articles (3662 from PubMed, 258 from CENTRAL, 5090 from WoS, 4546 from SCOPUS, and 3270 from EMBASE). Finally, seven studies were included in this systematic review and meta-analysis. *Figure 1* outlines the PRISMA flow diagram.

### Characteristics of included studies

We included seven studies: three RCTs^7^^,^^11^^,^^23^ and four observational studies.^12^^,^^24–26^ Our cohort included 9702 patients, with 4126 in the exercise arm and 5576 in the control arm. The three RCTs^7^^,^^11^^,^^23^ included 275 patients, 136 in the exercise group and 139 in the control group. The four observational studies^12^^,^^24–26^ included 9427 patients, 3990 in the exercise group and 5437 in the control group. Follow-up periods ranged from 3 months to 7 years. All studies included adult patients; however, Lampert et al^12^ included patients aged 8 to 80 years old. *Tables 1 and 2* detail the characteristics of the included studies and the included participants.

**Table 1.** Summary characteristics of the included studies

Study	Study design	Country	Total participants	Exercise details		Control	Main inclusion criteria	Exclusion criteria	Primary outcome	Composite outcome definition	Follow- up duration
Basu et al 2025 ^7^	Multicenter randomized controlled trial	United Kingdom	80	Exercise classes consisted of a warm-up, a circuit of alternating aerobic and resistance exercises, and a cool-down. At least 50% of the hour was dedicated to aerobic activities. Participants began exercising at high intensity at 70% of their heart rate reserve (HRR). Intensity progressed by 5% increments to 85% of HRR.	12-wk program, with 1 h supervised exercise twice weekly and 1-h home-based exercise per week	Usual care	Eligible patients were those with a diagnosis of HCM (LVWT ≥ 15 mm in the absence of abnormal loading conditions), aged 16–60 yr, NYHA I–II, able to exercise, and committed to the protocol-defined program duration	1) Competitive athletes; 2) history of exercise-induced syncope; 3) poorly controlled ventricular arrhythmias; 4) surgical myectomy; 5) severely reduced left ventricular (LV) ejection fraction (<35%); 6) LV outflow tract gradient ≥ 50 mm Hg at rest, following Valsalva maneuver or squatting; 7) awaiting or recent device implantation; 8) known coronary artery disease (lesion > 50% on coronary angiography/previous coronary intervention); 9) exercise limited by a noncardiac cause; 10) renal failure; 11) phenocopies of HCM; and 12) pregnancy.	A composite of arrhythmia-related events	A composite of arrhythmia-related events (cardiovascular death, cardiac arrest, appropriate or inappropriate ICD therapy, exercise-induced syncope, sVT, NsVT) and sustained atrial arrhythmias (≥30 s).	3 mo
Cavigli et al 2024 ^24^	Retrospective and prospective cohort study	Italy	13	Aerobic exercise, high and moderate-intensity	Suggested: 150–300 min/wk Frequency: Start with 2 sessions/wk, target 3–5 sessions/wk	Sedentary activity	Patients with a diagnosis of HCM aged between 18 and 55 yr	1) NYHA functional class III or IV; 2) impossibility of performing functional tests; 3) noncardiac causes of functional limitation; 4) septal reduction therapy or cardioverter-defibrillator implantation in the previous 3 months; 5) acute heart failure or hospitalization in the previous 3 months; 6) severe ventricular dysfunction, changes in therapy in the previous 3 months; and 7) pregnancy.	Effect of moderate-intensity exercise	NA	Mean (SD): 24 (12) mo
Gudmundsdottir et al 2024^11^	Multicenter, randomized controlled trial	Denmark	59	Aerobic and resistance exercise, moderate intensity, tailored to 60% of peak work capacity	1 h per session, 3 sessions/wk, 12 wk total	Usual care	Patients with HCM and LVWT ≥15 mm on echocardiography, no LVOT obstruction (i.e., gradient <30 mm Hg at rest or with provocation), age ≥ 18 yr, NYHA functional class I–III, stable clinical condition, and able and willing to participate in supervised exercise training	1) Peak LVOT gradient ≥30 mm Hg at rest or with Valsalva maneuver provocation, or ≥50 mm Hg immediately after exercise; 2) history of exercise-induced syncope or ventricular arrhythmias within the last year; 3) severe angina symptoms (Canadian Cardiovascular Society class III–IV); 4) hemodynamically severe valvular dysfunction; 5) <3 months after septal reduction therapy; 6) severe hypertension (systolic blood pressure > 200 mm Hg or diastolic >110 mm Hg); 7) patients already performing dedicated moderate- or high-intensity exercise >1 h weekly (determined according to ratings of self-reported exertion); 8) recent (within 1 month) changes in medication that may affect exercise capacity or hemodynamics (i.e., beta-blockers and calcium channel blockers); 8) conditions precluding the ability to exercise; 9) pregnancy or planned pregnancy; 10) any condition or situation that, in the investigator’s opinion, could put the subject at significant risk, confound the study results, or interfere significantly with the subject’s participation in the study.	PCWP during mild exercise (25 W) from baseline to week 12	A composite of death, aborted SCD, sVT, ICD shock, and any admissions.	3 mo
Kwon et al 2020 ^26^	Retrospective cohort study	South Korea	7666	More than usual physical activity (moderate to vigorous-intensity), aerobic exercise	Participants performed moderate-intensity physical activity, such as brisk walking, light sports, and casual cycling (5 METs 30 min/session). Participants engaged regularly in vigorous-intensity physical activity, e.g., running, aerobics, mountain climbing (8 METs 20 min/session)	Sedentary and usual activity	Adult patients with HCM with diagnostic codes I42.1 or I42.2 were registered in the RID program (confirmed by imaging and expert review) and underwent health checkups within 1 yr of diagnosis	1) Not registered as HCM in the RID program; 2) did not undergo health checkups within 1 yr after the diagnosis of HCM; 3) missing data on the study variables; 4) reported no physical activity at all in the health examination questionnaires due to the concern that such individuals may have severe comorbidities preventing them from performing physical activity	Mortality	NA	Mean (SD): 5.3 (2.0) yr
Lampert et al 2023 ^12^	Prospective cohort study	USA, UK, Canada, Australia, and New Zealand	1660	Moderate to vigorous exercise	Vigorous: Participation in at least one activity at a MET intensity level of ≥6.0 for ≥60 h per yr Moderate: Participation in activities at a MET intensity level of ≥4.0 but <6.0 for ≥60 h per yr	Sedentary activity	Patients aged 8–60 yr, diagnosed with overt HCM or genotype positive / phenotype negative, able to provide informed consent, able to complete online/phone questionnaires, and without exclusionary comorbidities or conditions	1) Patients with conditions precluding vigorous exercise (advanced HCM-related symptoms, i.e., NYHA class III or IV, or non–HCM-related conditions); 2) patients with left ventricular hypertrophy due to syndromic conditions or infiltrative disease; 3) individuals unable to complete online or phone questionnaires due to language or cognitive barriers	Composite of death, resuscitated sudden cardiac arrest, arrhythmic syncope, and appropriate shock from an ICD	Composite of death, resuscitated SCD, arrhythmic syncope, and appropriate shock from an ICD	3 yr
Pelliccia et al 2020 ^25^	Retrospective cohort study	Italy	88	High-intensity exercise	≥3 sessions/wk, ≥2 h/session, including high-intensity exercise bouts, for a global training total of ≥6 and up to 14 h/wk	HCM-detrained (sedentary or engaged in leisure-time and only occasional exercise programs, <6 h/wk	Adult athletes diagnosed with HCM (LVWT ≥15 mm or 13–14 mm with family/genetic/ECG evidence) and engaged in competitive sport at inclusion	NA	Safety outcomes	NA	7 yr
Saberi et al 2017 ^23^	Multicenter, randomized controlled trial	USA	136	Aerobic exercise, moderate-intensity exercise	Began at 20 min/session; increased by 5–10 min/wk; targeted 60 min/session; total duration: 16 wk	Usual activity	Patients aged 18–80 yr with LVWT ≥15 mm (or 13–15 mm with suggestive features)	1) History of exercise-induced syncope or ventricular arrhythmias; 2) medically refractory LVOT obstruction being evaluated for septal reduction therapy; 3) <3 mo after septal reduction therapy or ICD placement; 4) history of hypotensive response with exercise testing (>20 mm Hg decrease of systolic blood pressure from baseline or an initial increase in systolic blood pressure followed by a decrease of >20 mm Hg); 5) clinical decompensation in the previous 3 mo, defined as NYHA class IV congestive heart failure symptoms or Canadian Cardiovascular Society class IV angina symptoms; 6) left ventricular ejection fraction <55% by echocardiography; 7) life expectancy <12 months; 8) pregnant or planned pregnancy; 9) inability to exercise owing to noncardiovascular limitations; and 10) unwillingness to refrain from competitive sports, burst activity, or heavy isometric exercise for the duration of the study.	Change in peak VO_2_	A composite of death, aborted SCD, appropriate ICD shocks, or sVT	4 mo

HCM indicates hypertrophic cardiomyopathy; ICD, implantable cardioverter defibrillator; LVEF: Left ventricular ejection fraction; LVOT, left ventricular outflow tract; LVWT, left ventricular wall thickness; MET, metabolic equivalent of tasks; NA, not available; NsVT, nonsustained ventricular tachycardia; NYHA, New York Heart Association; PCWP, pulmonary capillary wedge pressure; peak VO_2_, peak oxygen consumption; RID, rare intractable diseases; SCD, sudden cardiac death; sVT, sustained ventricular tachycardia.

**Table 2.** Baseline characteristics of the participants

Study ID	Patients: N		Male: N (%)		Age (years): Mean (SD)		BMI (kg/m^2^): Mean (SD)		LVEF (%): Mean (SD)		Peak VO_2_ (mL/kg/min): Mean (SD)		NYHA: N (%)				Comorbidities: N (%)								Medications: N (%)			
													I		**II-III**													
	EX	CO	EX	CO	EX	CO	EX	CO	EX	CO	EX	CO	EX	CO	EX	CO	Hypertension		Diabetes		AF		Stroke/TIA		Beta-blocker		CCBs	
																	EX	CO	EX	CO	EX	CO	EX	CO	EX	CO	EX	CO
Basu et al 2025^7^	40	40	36 (90)	31 (77.5)	48 (7.9)	44 (8.9)	27.97 (3.7)	27.83 (5.2)	65 (7.69)	65 (7.69)	28.73 (9.15)	29.07 (10.38)	NA	NA	NA	NA	12 (30)	14 (35)	2 (5)	1 (2.5)	NA	NA	1 (2.5)	0 (0)	13 (32.5)	9 (22.5)	9 (22.5)	7 (17.5)
Cavigli et al 2024^24^	10	3	NA	NA	NA	NA	NA	NA	NA	NA	NA	NA	NA	NA	NA	NA	NA	NA	NA	NA	NA	NA	NA	NA	NA	NA	NA	NA
Gudmundsdottir et al 2024^11^	29	30	21 (72)	22 (73)	56.3 (11.3)	59.9 (12.9)	29.3 (4.8)	26.6 (7.0)	59 (9)	58 (9)	20.2 (6.4)	21.1 (7.0)	4 (14)	9 (30)	25 (68)	21 (70)	12 (41)	13 (43)	1 (3.4)	3 (10)	8 (28)	6 (20)	3 (10)	1 (3.3)	18 (62)	15 (50)	1 (3.4)	3 (10)
Kwon et al 2020^26^	2545	5121	1974 (77.6)	3478 (67.9)	59.3 (11.4)	59.6 (12.4)	25.1 (2.9)	25.2 (3.2)	NA	NA	NA	NA	NA	NA	NA	NA	1711 (67.3)	3425 (66.9)	471 (18.5)	932 (18.2)	390 (15.3)	791 (15.4)	NA	NA	NA	NA	NA	NA
Lampert et al 2023 (vigorous intensity)^12^	699	252	467 (66.8)	118 (46.8)	36.1 (15.3)	41.5 (13.8)	NA	NA	66.1 (7.1)	65.2 (8.6)	NA	NA	NA	NA	NA	NA	NA	NA	NA	NA	NA	NA	NA	NA	NA	NA	NA	NA
Lampert et al 2023 (moderate intensity)^12^	709		400 (56.4)		40.2 (13.9)		NA		66.6 (7.3)		NA	NA	NA		NA		NA		NA		NA		NA		NA		NA	
Pelliccia et al 2020^25^	27	61	25 (93)	56 (92)	32.67 (25.05)	29.67 (15.9)	NA	NA	65 (4.6)	65 (4.6)	NA	NA	NA	NA	NA	NA	NA	NA	NA	NA	NA	NA	NA	NA	NA	NA	NA	NA
Saberi et al 2017^23^	67	69	38 (56.7)	41 (59.4)	50.5 (13.2)	50.0 (13.5)	30.6 (5.6)	31.4 (5.3)	70.6 (4.0)	70.8 (4.3)	21.3 (6.3)	22.5 (7.2)	44 (65.7)	46 (66.7)	23 (34.3)	23 (33.3)	14 (20.9)	16 (23.2)	4 (6.0)	5 (7.2)	14 (20.9)	8 (11.6)	NA	NA	41 (61.2)	51 (73.9)	15 (22.4)	14 (20.3)

AF indicates atrial fibrillation; BMI, body mass index; CCBs, calcium channel blockers; CO, control group; EX, exercise group; NA, not available; SD, standard deviation; TIA, transient ischemic stroke.

### Risk of bias and certainty of evidence

**Figure 2.**
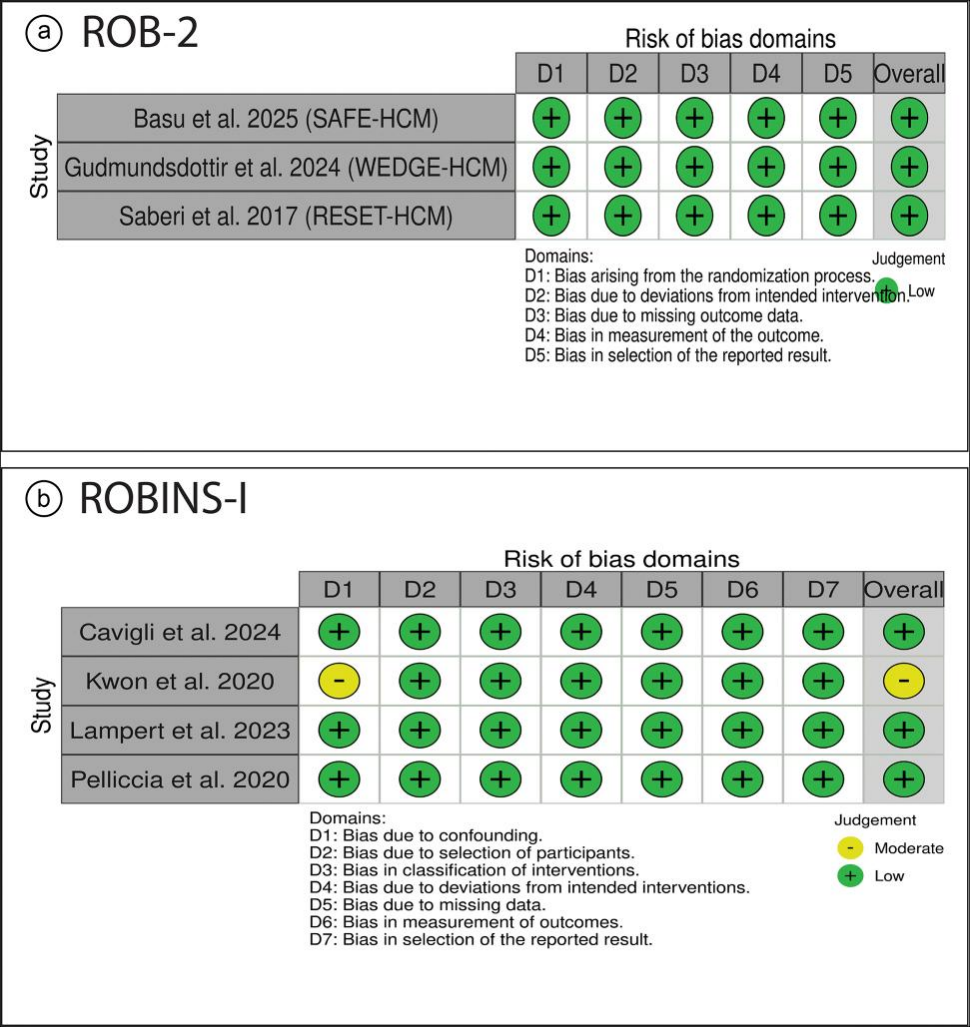
Quality assessment of the risk of bias (ROB) for the included trials: **(a)** ROB assessment for randomized controlled trials (ROB-2 tool); **(b)** ROB assessment for nonrandomized trials (ROBINS-I).

Based on application of the RoB 2.0 and ROBINS-I tools, all included RCTs had a low risk of bias.^7^^,^^11^^,^^23^ However, one observational study had moderate concerns;^26^ the remaining observational studies had a low risk of bias^12^^,^^24^^,^^25^ *(Figure 2).*

### Cardiorespiratory fitness

**Figure 3.**
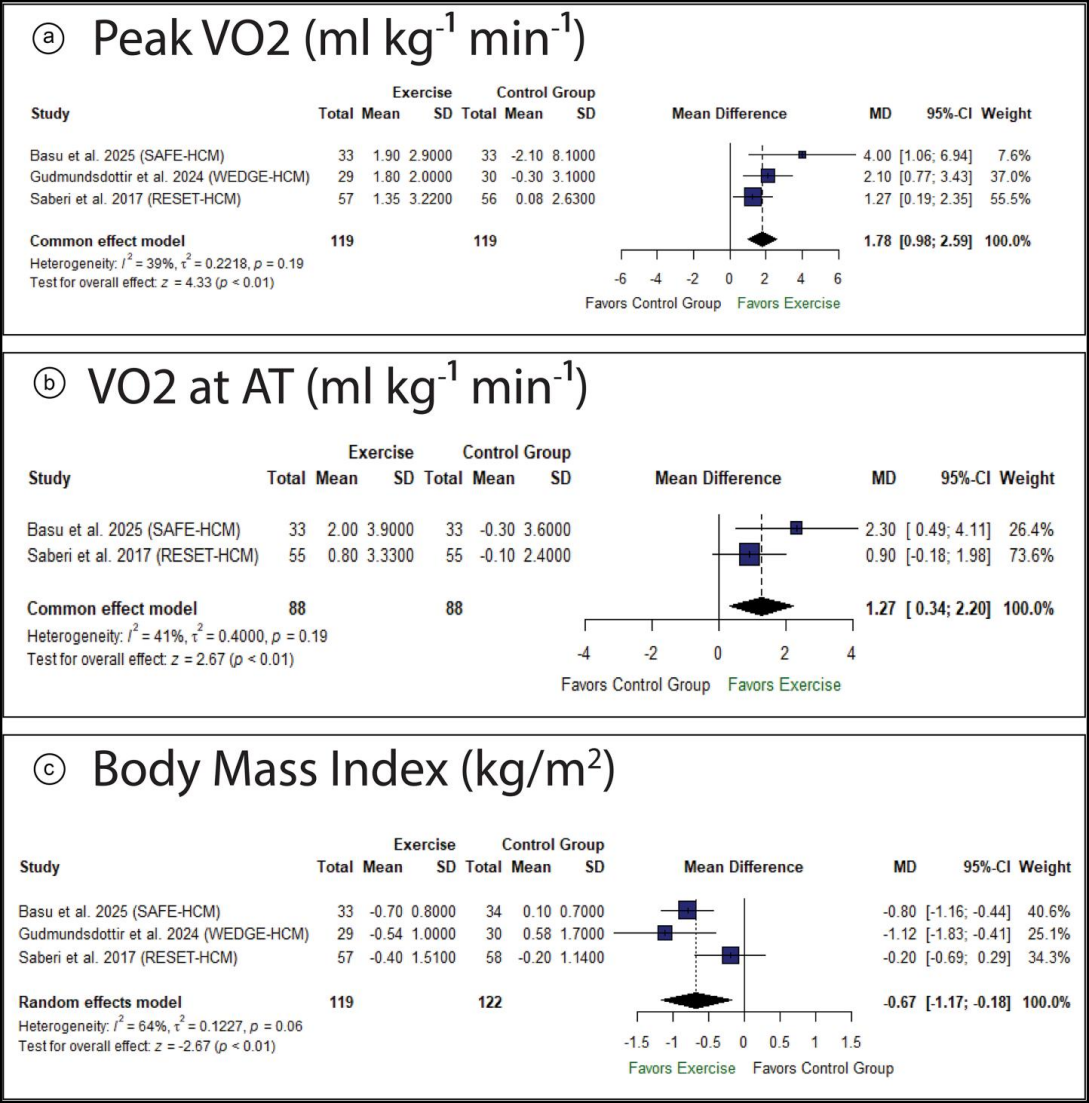
Forest plot of **(a)** mean change of peak oxygen uptake (peak VO_2_); **(b)** VO_2_ at anaerobic threshold; and **(c)** body mass index. CI indicates confidence interval; MD, mean difference.

Pooling three RCTs^7^^,^^11^^,^^23^ with 238 patients, exercise was significantly associated with increased peak VO_2_ (mL∙kg^−1^∙min^−1^) (MD: 1.78; 95% CI [0.98, 2.59]; *P* < 0.01) compared to the control group *(Figure 3a)*. Subgroup analysis showed no significant difference between moderate- and high-intensity exercise protocols (*P* = 0.12) *(Supplemental Figure 1)*. Pooling two RCTs^7^^,^^23^ with 176 patients, exercise was significantly associated with increased VO_2_ at AT (mL∙kg^−1^∙min^−1^) (MD: 1.27; 95% CI [0.34, 2.20]; *P* < 0.01) compared to the control group *(Figure 3b)*. The pooled studies were homogenous in peak VO_2_ (I^2^= 39%, *P* = 0.19) and VO_2_ at AT (I^2^= 41%, *P* = 0.19).

### Secondary outcomes

#### Body mass index

Pooling three RCTs^7^^,^^11^^,^^23^ with 241 patients, exercise was significantly associated with reduced BMI (kg/m^2^) (MD: −0.67; 95% CI [−1.17, −0.18]; *P* < 0.01) compared to the control group *(Figure 3c)*. Subgroup analysis showed no significant difference between moderate- and high-intensity exercise protocols (*P* = 0.72) *(Supplemental Figure 2)*. The pooled studies were heterogeneous (I^2^= 64%, *P* = 0.06), which was best resolved by excluding Saberi et al^23^ (I^2^= 0%) *(Supplemental Figure 3)*.

#### Left ventricular wall thickness

**Figure 4.**
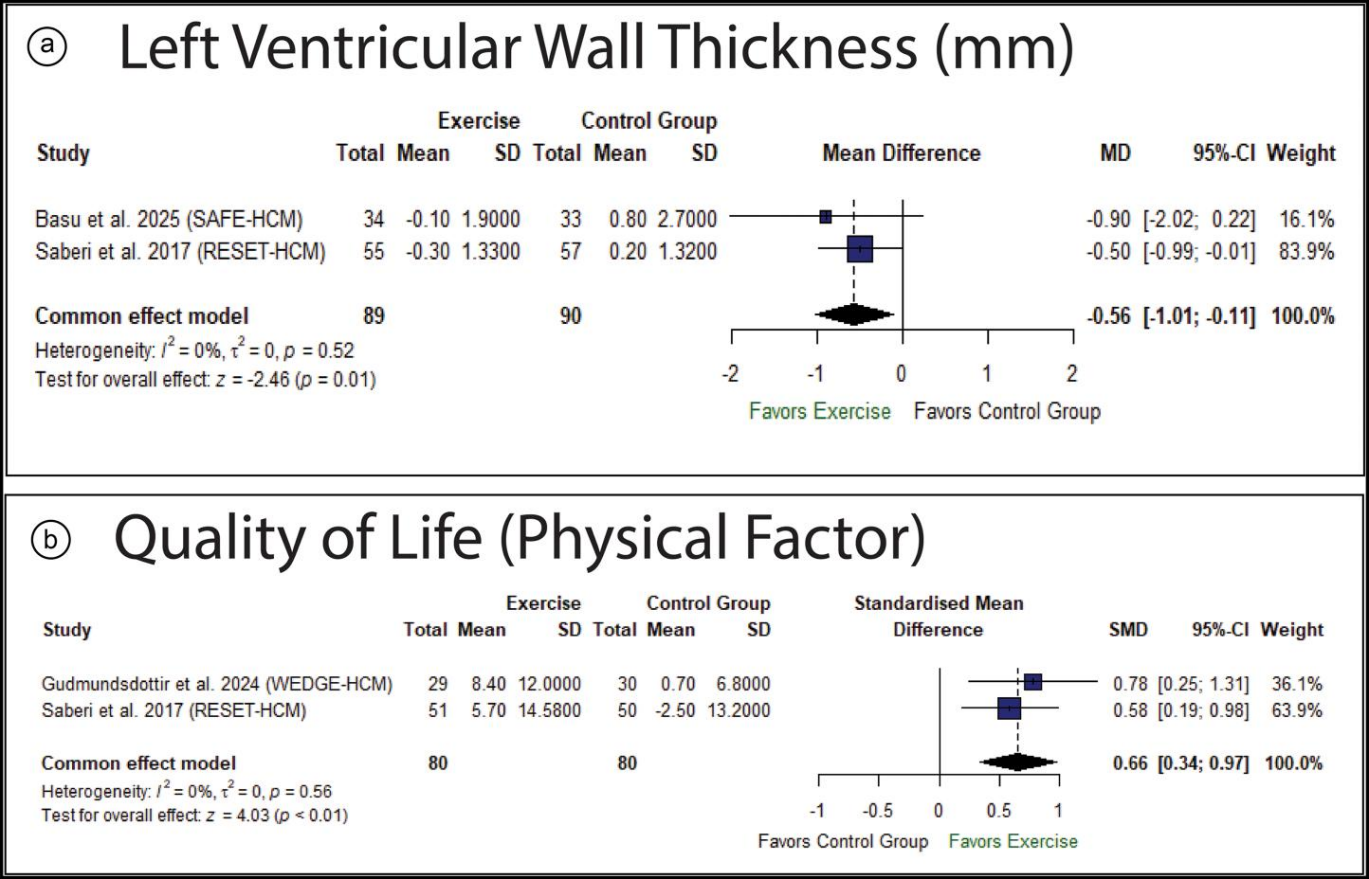
Forest plot of **(a)** left ventricular wall thickness and **(b)** quality of life (physical factor). CI indicates confidence interval; MD, mean difference, SMD, standardized mean difference.

Pooling two RCTs^7^^,^^23^ with 179 patients, exercise was significantly associated with reduced LVWT (mm) measured by echocardiography (MD: −0.56; 95% CI [−1.01, −0.11]; *P* = 0.01) compared to the control group *(Figure 4a)*. The pooled studies were homogenous (I^2^= 0%, *P* = 0.52).

#### Postexercise left ventricular outflow tract pressure gradient

Pooling two RCTs^11^^,^^23^ with 163 patients, there was no significant difference between the exercise and control groups in postexercise LVOT pressure gradient (mm Hg) (MD: 2.26; 95% CI [−1.87, 6.40]; *P* = 0.28) *(Supplemental Figure 4)*. The pooled studies were homogenous (I^2^= 0%, *P* = 0.38).

#### Exercise time

Pooling two RCTs^7^^,^^23^ with 144 patients, there was no significant difference in exercise time (seconds) between the exercise and control groups (MD: 54.60; 95% CI [−26.86, 136.07]; *P* = 0.19) *(Supplemental Figure 5)*. The pooled studies were heterogeneous (I^2^= 66%, *P* = 0.09). Sensitivity analysis was not applicable.

#### E/e′ ratio

Pooling two RCTs^7^^,^^11^ with 120 patients, there was no significant difference between the exercise and control groups in E/e′ ratio (MD: 0.28; 95% CI [−0.57, 1.12]; *P* = 0.52) *(Supplemental Figure 6)*. The pooled studies were homogenous (I^2^= 0%, *P* = 0.85).

#### E/A

Pooling two RCTs^7^^,^^11^ with 120 patients, there was no significant difference between the exercise and control groups in E/A (MD: 0.02; 95% CI [−0.17, 0.20]; *P* = 0.86) *(Supplemental Figure 7)*. The pooled studies were homogenous (I^2^= 0%, *P* = 0.70).

#### N-terminal pro-B-type natriuretic peptide

Pooling two RCTs^7^^,^^11^ with 113 patients, there was no significant difference between the exercise and control groups in NT-ProBNP (ng/L) (MD: 45.16; 95% CI [−16.06, 106.37]; *P* = 0.15) *(Supplemental Figure 8)*. The pooled studies were homogenous (I^2^= 36%, *P* = 0.21).

#### Quality of life

Pooling two RCTs^7^^,^^11^ with 160 patients, exercise was significantly associated with increased QoL physical factor (SMD: 0.66; 95% CI [0.34, 0.97]; *P* < 0.01) compared to the control group *(Figure 4b)*. However, pooling two RCTs^11^^,^^23^ with 123 patients, there was no significant difference between the exercise and control groups in general QoL (SMD: 0.24; 95% CI [−0.39, 0.86]; *P* = 0.46) *(Supplemental Figure 9)*. The pooled studies were homogeneous in QoL physical factor (I^2^= 0%, *P* = 0.56), while heterogeneous in QoL general (I^2^= 67%, *P* = 0.08). Sensitivity analysis was not applicable. This outcome was mainly based on RCTs.

### Safety outcomes

**Figure 5.**
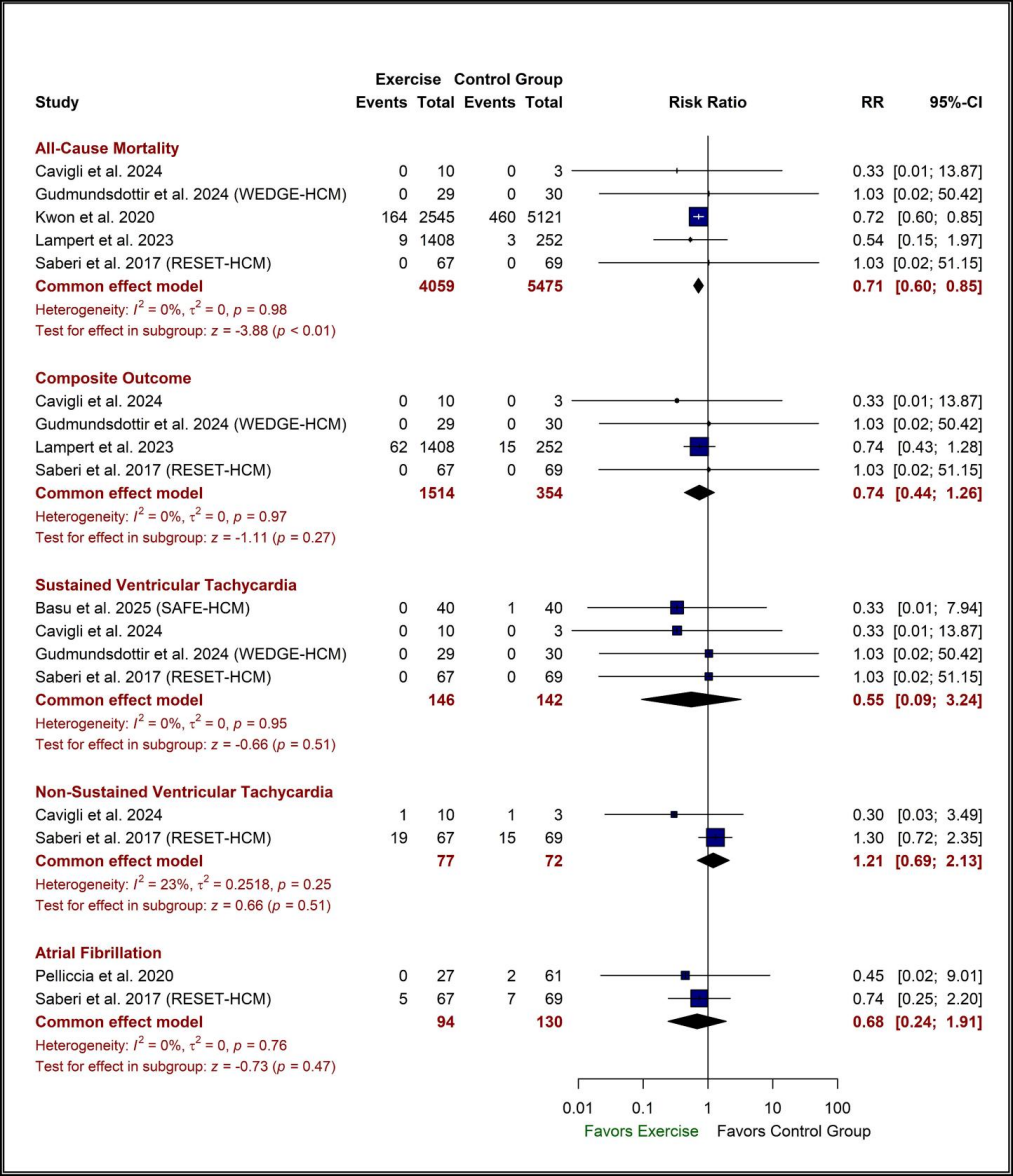
Forest plot of safety outcomes. CI indicates confidence interval; RR, risk ratio.

With a maximum follow-up of 7 years across the included studies, exercise was significantly associated with a lower incidence of all-cause mortality (RR: 0.71; 95% CI [0.60, 0.85]; *P* < 0.01) compared to the control group *(Figure 5)*. However, there was no significant difference between the exercise and control groups in composite outcome (RR: 0.74; 95% CI [0.44, 1.26]; *P* = 0.27) *(Figure 5)*, sustained ventricular tachycardia (sVT) (RR: 0.55; 95% CI [0.09, 3.24]; *P* = 0.51) *(Figure 5)*, nonsustained ventricular tachycardia (NsVT) (RR: 1.21; 95% CI [0.69, 2.13]; *P* = 0.51) *(Figure 5)*, atrial fibrillation (RR: 0.68; 95% CI [0.24, 1.91]; *P* = 0.47) *(Figure 5)*, cardiac arrest/SCD (RR: 0.70; 95% CI [0.18, 2.72]; *P* = 0.61) *(Supplemental Figure 10)*, syncope (RR: 0.92; 95% CI [0.52, 1.64]; *P* = 0.79) *(Supplemental Figure 10)*, palpitations (RR: 0.70; 95% CI [0.15, 3.30]; *P* = 0.65) *(Supplemental Figure 10)*, and ICD implantation (RR: 1.45; 95% CI [0.47, 4.50]; *P* = 0.52) *(Supplemental Figure 10)*.

The pooled studies were homogeneous in all-cause mortality (I^2^= 0%, *P* = 0.98), composite outcome (I^2^= 0%, *P* = 0.97), sVT (I^2^= 0%, *P* = 0.95), NsVT (I^2^= 23%, *P* = 0.25), atrial fibrillation (I^2^= 0%, *P* = 0.76), cardiac arrest/SCD (I^2^= 0%, *P* = 0.99), syncope (I^2^= 0%, *P* = 0.43), palpitations (I^2^= 16%, *P* = 0.27), and ICD implantations (I^2^= 0%, *P* = 0.86). Subgroup analysis showed no significant difference between moderate- and high-intensity exercise protocols in safety outcomes (*P* > 0.1) *(Supplemental Figures 11 and 12)*. Subgroup analysis comparing RCTs and observational studies showed that, when pooling only observational studies, exercise was significantly associated with a lower incidence of all-cause mortality (RR: 0.71, 95% CI [0.60, 0.85]; *P* < 0.01) compared with the control group. However, upon including only the RCTs, there was no significant difference in all-cause mortality between the exercise and control groups (RR: 1.03; 95% CI [0.07, 16.22]; *P* = 0.98) *(Supplemental Figure 13)*. Additionally, subgroup analysis comparing RCTs and observational studies showed no significant difference in the composite outcome between the exercise and control groups in either group *(Supplemental Figure 14)*.

## DISCUSSION

This systematic review and meta-analysis provide evidence that exercise interventions in patients with HCM are safe and beneficial. Our findings challenge longstanding restrictions on physical activity in HCM and support a growing consensus that, when carefully prescribed and supervised, exercise should be integrated into routine care for these patients.

We found that exercise was significantly associated with improvements in CRF (peak VO_2_ MD: 1.78 mL∙kg^−1^∙min^−1^, and VO_2_ at AT: MD: 1.27 mL∙kg^−1^∙min^−1^) compared to the control group. However, the improvement was modest. Even small improvements can carry significant prognostic value; each 1 mL∙kg^−1^∙min^−1^ increase was associated with lower all-cause, cardiovascular, and cancer mortality.^27^^,^^28^ Specifically, in those with HCM, exercise intolerance is a key determinant of symptom burden and QoL; therefore, even modest improvements in CRF may translate into clinically perceptible benefits. Favorable hemodynamic adaptations may mediate the observed gains in exercise capacity, as several trials reported enhanced peripheral oxygen utilization and reduced left ventricular filling pressures during exertion.^11^^,^^29^ These improvements were consistently observed across multiple studies involving moderate- or high-intensity training in patients with HCM. While the magnitude of improvement varied, the direction of effect was uniform, supporting the role of supervised exercise training in improving CRF in this population.

Despite these functional gains, no significant differences were observed in postexercise LVOT gradient, E/e′ ratio, or E/A ratio compared to the control group. This suggests that exercise primarily improves CRF rather than altering diastolic indices or dynamic outflow obstruction. Several factors can explain this. First, atrial fibrillation was inconsistently reported across studies, and its presence can significantly affect parameters such as E/A and E/e′, potentially introducing variability. Second, the number of studies reporting these outcomes was limited to two RCTs per outcome, and the sample sizes were small, reducing statistical power. Finally, the relatively short duration of the exercise interventions and follow-up (up to 4 months) may have been insufficient to induce measurable changes in these structural and hemodynamic parameters.

Additionally, our study showed that exercise was significantly associated with reduced LVWT, measured by echocardiography in the two studies pooled: Basu et al (SAFE-HCM)^7^ and Saberi et al (RESET-HCM).^23^ In HCM, an increase in myocardial fibrosis is significantly associated with lower CRF.^30^ Weissler-Snir et al and Rowin et al reported no evidence of adverse remodeling or disease progression in physically active patients with HCM.^31^^,^^32^ However, Saberi et al (RESET-HCM)^23^ showed a significant subgroup difference between exercise and usual activity when evaluating maximal LV thickness by echocardiography, with a between-group difference of −0.6 mm (*P* = 0.02); no significant difference was observed when the cardiac magnetic resonance imaging (MRI) was used.

Exercise was also associated with a modest but statistically significant reduction in BMI (MD: −0.67 kg/m^2^), aligning with prior observations that physically active patients with HCM tend to exhibit fewer metabolic comorbidities. Obesity is highly prevalent in HCM and has been independently linked to increased left ventricular mass, diastolic dysfunction, and a greater symptom burden, as highlighted in reviews by Finocchiaro et al and Zaromytidou et al.^33^^,^^34^ Given that patients with HCM often reduce physical activity after the diagnosis, either due to symptoms or medical advice, this sedentary behavior may contribute to obesity-related hemodynamic deterioration, including elevated left ventricular filling pressures. The current findings underscore the potential value of exercise in mitigating modifiable cardiovascular risk factors and improving functional status in this population.

Moreover, exercise was associated with significant improvements in the physical component of QoL (SMD: 0.66), whereas no statistically significant difference was observed in the general QoL. The physical QoL component primarily reflects symptom burden, functional limitations, and the ability to perform daily activities. In patients with HCM, improvement in this domain is consistent with the observed increase in CRF and likely represents a meaningful functional benefit. The absence of change in overall QoL may reflect the broader, multifactorial nature of global QoL measures, which incorporate psychological and social dimensions that short-term exercise interventions may not directly modify.

Perhaps most critically, this analysis found that exercise participation was associated with a lower incidence of all-cause mortality. However, subgroup analysis of RCTs versus observational studies showed that, when pooling only the observational studies, exercise was significantly associated with a lower incidence of all-cause mortality compared to the control group. However, when only RCTs were included, there was no significant difference in all-cause mortality between the exercise and control groups. This indicates that observational studies mainly drove the evidence for a reduction in all-cause mortality and should be interpreted with caution. Additionally, there was no significant difference in adverse safety events, including NsVT, syncope, palpitations, or SCD, between the exercise and control groups. In contrast, Albulushi et al^35^ reported no significant difference in mortality between exercise and control groups. Unlike their analysis, our study included more studies and was restricted to those specifically comparing exercise with usual care, usual activity, or a sedentary lifestyle. These findings are consistent with several studies reporting no increased risk of arrhythmia, SCD, or ICD therapy in patients with HCM who engage in recreational or competitive exercise.^12^^,^^36–38^ Moreover, a review by Saberi and Day has questioned the historical attribution of exertional SCD to HCM in the absence of high-risk features, suggesting that prior risk estimates may have been overstated.^39^

### Clinical implications and future research

This study suggests that exercise may be safe and beneficial for patients with HCM. Both the American Heart Association/American College of Cardiology HCM and European Society of Cardiology sports cardiology guidelines endorse a more liberal approach to physical activity in this population, moving away from categorical exercise restrictions toward individualized recommendations based on shared decision-making and comprehensive risk assessment.^15^^,^^40^ Our meta-analysis showed that individualized, supervised exercise appears to improve CRF, BMI, and LVWT in patients with HCM without a detectable increase in adverse events or mortality. In routine clinical practice, exercise prescriptions should be carefully tailored to the patient’s phenotypic expression, symptom burden, arrhythmic risk profile, and comorbid conditions.^24^ CPET plays a central role in defining baseline limitations, guiding exercise intensity, and identifying latent obstruction or chronotropic incompetence.^41^

However, larger, longer trials are needed to assess the long-term effects of different exercise modalities, including resistance training and high-intensity interval training, across diverse populations with HCM. Integrating cardiac MRI, genotyping, and wearable cardiac monitoring will enhance our ability to stratify risk and optimize exercise prescription. Cardiac MRI will more accurately assess the impact of exercise on LVWT than echocardiography. Special emphasis should be placed on evaluating exercise in younger patients, those with sarcomeric mutations, and those with varying degrees of obstruction.

### Strengths and limitations

To our knowledge, this is the first comprehensive meta-analysis to assess the efficacy and safety of exercise interventions in patients with HCM. However, there are several limitations to this meta-analysis: 1) the low number of included RCTs; 2) the short follow-up period of 3 to 4 months of the included RCTs, which our paper used solely for the primary and secondary efficacy outcomes; 3) heterogeneity in exercise protocol duration, intensity, and supervision across included studies; 4) small cohorts and short follow-ups in the included RCTs, limiting the ability to evaluate long-term safety; 5) exclusion of high-risk patients, such as those with severe obstruction or prior SCD, reducing generalizability; 6) inconsistencies in arrhythmia detection and adverse event definitions, which may have influenced the accuracy of pooled safety estimates; and 7) all-cause mortality results driven mainly by observational studies. In addition, pooled LVWT outcomes were measured by echocardiography; however, when Saberi et al (RESET-HCM)^23^ assessed LVWT by cardiac MRI, there was no significant difference. This necessitates further RCTs with a large sample size and longer follow-up, investigating the impact of exercise on LVWT by more accurate imaging, such as cardiac MRI.

## CONCLUSION

This study suggests that structured exercise interventions are safe and associated with improvements in CRF, LVWT, BMI, and QoL physical factor in patients with HCM, without increasing the risk of arrhythmias. Although a lower mortality rate was observed, this finding was mainly driven by observational studies and should therefore be interpreted with caution. These findings support a transition from historic exercise restriction toward a more nuanced, individualized approach guided by shared decision-making and clinical risk profiling. In the era of precision cardiology, exercise should be regarded not as a risk to be avoided, but as a therapeutic tool to be prescribed. Future large-scale RCTs are warranted to confirm these findings.

## Supplementary Material

Supplemental Material
