# Supplementary material for: Efficacy and safety of exercise for hypertrophic cardiomyopathy: a systematic review and meta-analysis
Source: Proc (Bayl Univ Med Cent). 2026 May 18;39(4):669–81. doi: 10.1080/08998280.2026.2667666 (PMC13271290; doi:10.1080/08998280.2026.2667666)
Supplement: Supplemental Material [file UBMC_A_2667666_SM0881.docx]

**Title.**

**Efficacy and Safety of Exercise for Hypertrophic Cardiomyopathy: A Systematic Review and Meta-analysis.**

**Running Title.**

Exercise for Hypertrophic Cardiomyopathy.

**Authors.**

Ahmed Mazen Amin, MD^1^; Wafaa Shehada, MD^2^; Mohamed Elgebaly, MBBCh^3^; Dina Ayman, MBBCh^3^; Mahmoud Mahmoud Ibrahim, MD^4,5^; Basel Abdelazeem, MD^6^; Kevin Felpel, DO^6^.

**Affiliations.**

1. Faculty of Medicine, Mansoura University, Mansoura, Egypt.
2. Faculty of Medicine, Islamic University of Gaza, Gaza, Palestine.
3. Faculty of Medicine, Beni-Suef University, Beni-Suef, Egypt.
4. McLaren Health Care, Flint, Michigan, USA.
5. Michigan State University, East Lansing, Michigan, USA.
6. Department of Cardiology, West Virginia University, Morgantown, West Virginia, USA.

**Contents:**

**Figures.**Figure S1: Subgroup analysis for peak VO_2_ regarding exercise intensity protocols.

Figure S2: Subgroup analysis for body mass index (BMI) regarding exercise intensity protocols.

Figure S3: Sensitivity analysis of BMI.

Figure S4: Forest plot of post-exercise left ventricular outflow tract pressure gradient.

Figure S5: Forest plot of exercise time.

Figure S6: Forest plot of E/e′ Ratio.

Figure S7: Forest plot of E/A.

Figure S8: Forest plot of N-Terminal Pro-B-Type Natriuretic Peptide.

Figure S9: Forest plot of quality of life general.

Figure S10: Forest plot of safety outcomes.

Figure S11: Subgroup analysis for all-cause mortality regarding exercise intensity protocols.

Figure S12: Subgroup analysis for composite outcome regarding exercise intensity protocols.

Figure S13: Subgroup analysis for all-cause mortality regarding study design.

Figure S14: Subgroup analysis for composite outcome regarding study design.

**Tables.**Table S1: Search strategy.

**Figure S1: Subgroup analysis for peak VO_2_ regarding exercise intensity protocols.**


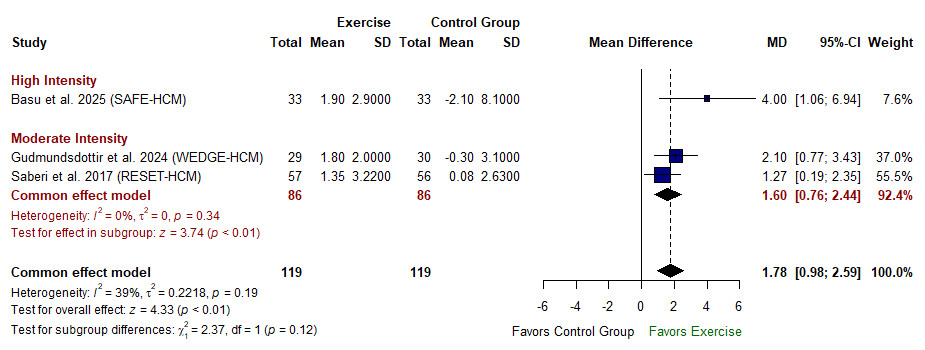


**Figure S2: Subgroup analysis for body mass index (BMI) regarding exercise intensity protocols.**


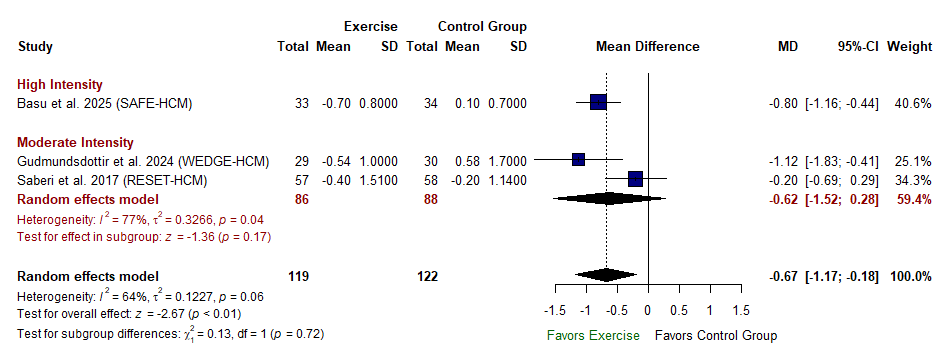


**Figure S3: Sensitivity analysis of BMI.**


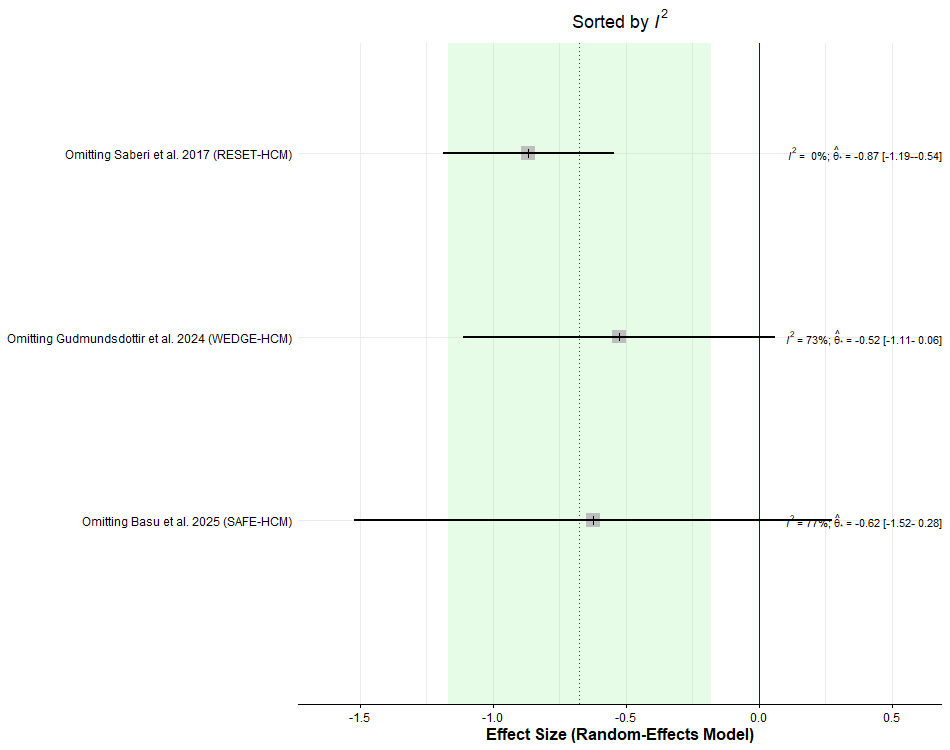


**Figure S4: Forest plot of post-exercise left ventricular outflow tract pressure gradient.**


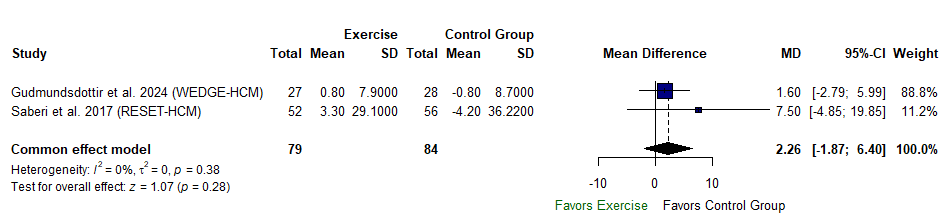


**Figure S5: Forest plot of exercise time.**


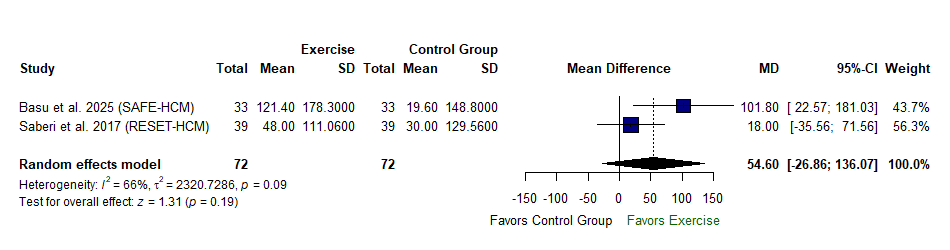


**Figure S6: Forest plot of E/e′ Ratio.**


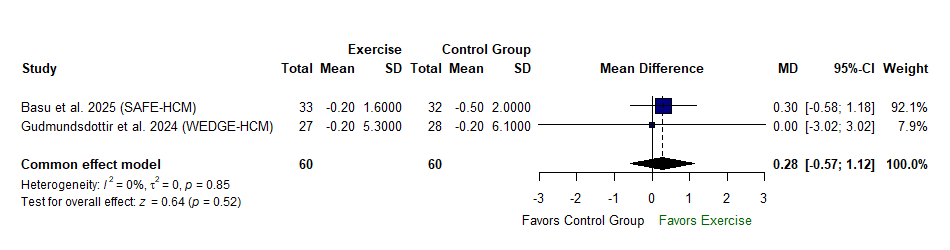


**Figure S7: Forest plot of E/A.**


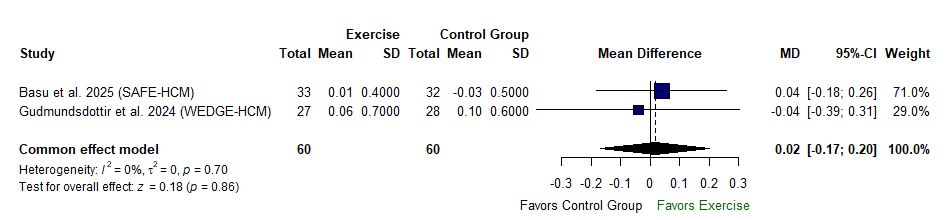


**Figure S8: Forest plot of N-Terminal Pro-B-Type Natriuretic Peptide.**


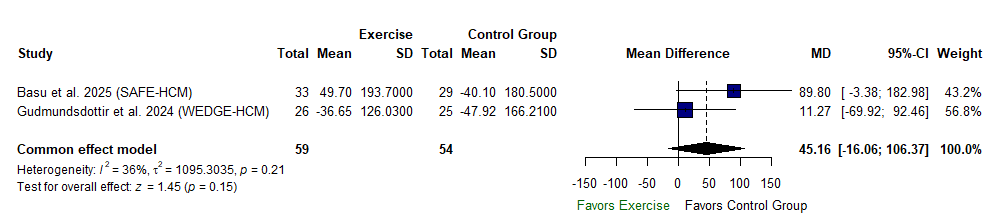


**Figure S9: Forest plot of quality of life general.**


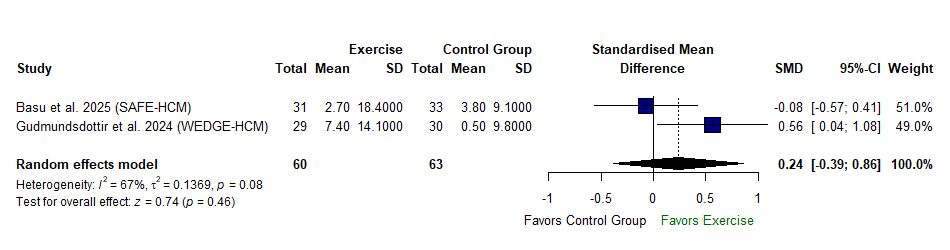


**Figure S10: Forest plot of safety outcomes**.


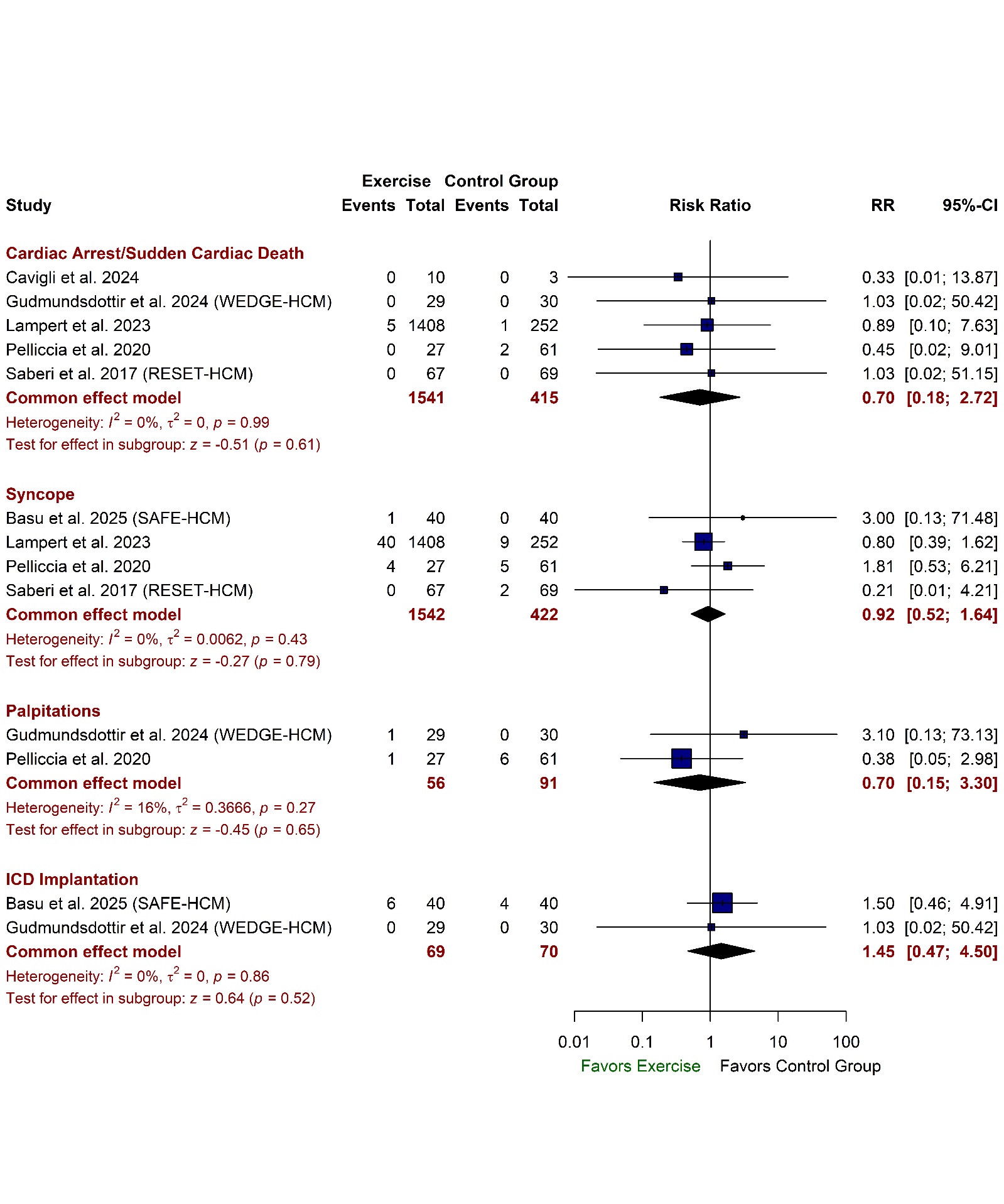


**Figure S11: Subgroup analysis for all-cause mortality regarding exercise intensity protocols.**

**
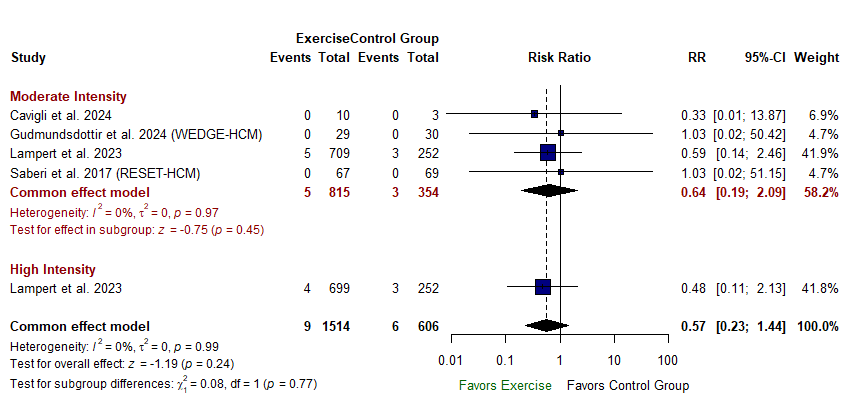
**

**Figure S12: Subgroup analysis for composite outcome regarding exercise intensity protocols.**

**
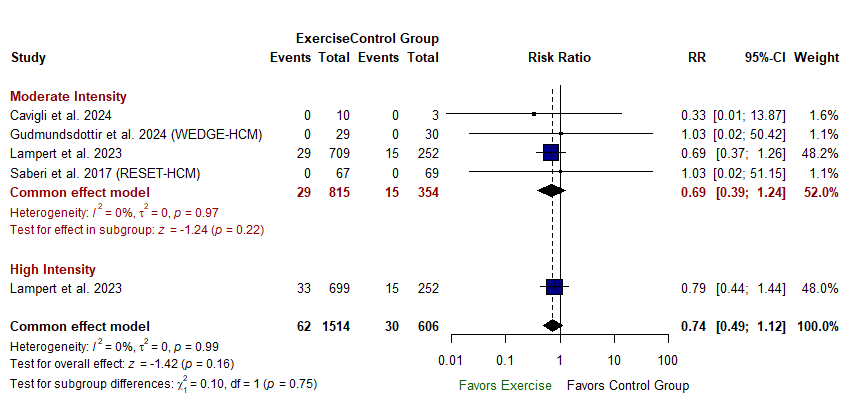
**

**Figure S13: Subgroup analysis for all-cause mortality regarding study design.**

**
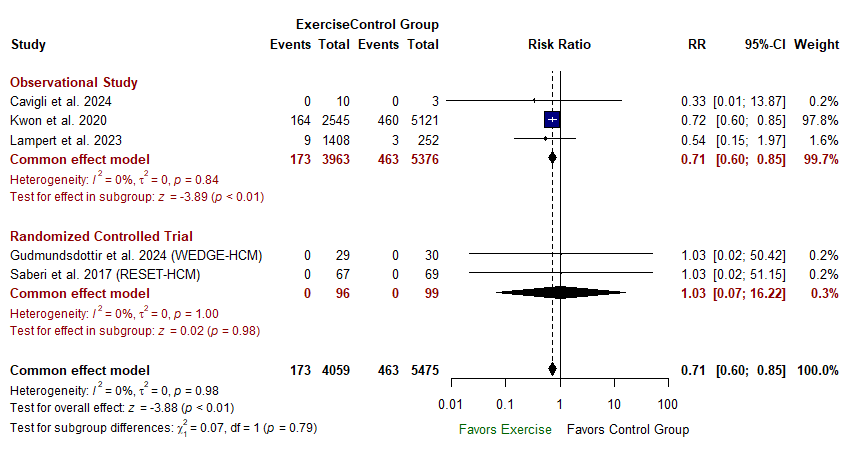
**

**Figure S14: Subgroup analysis for composite outcome regarding study design.**

**
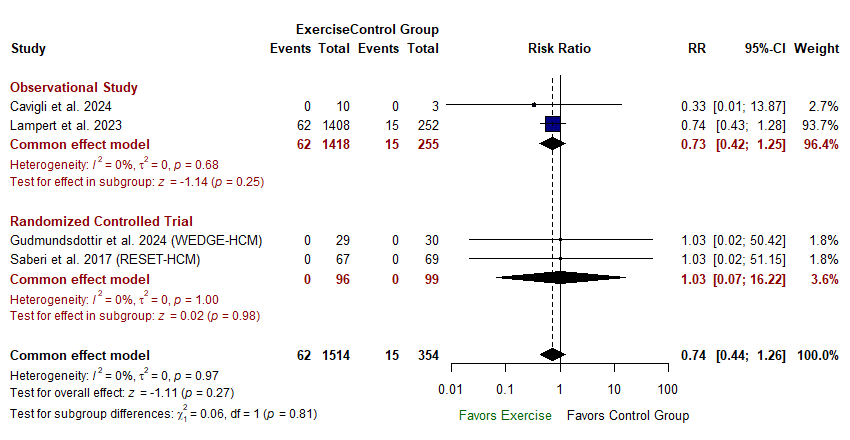
**

**Table S1: Search strategy.**

| **Database** | **Search Terms** | **Search Field** | **Search Results** |
| --- | --- | --- | --- |
| Pubmed | (Exercise* OR "Physical Activit*" OR walking OR sport* OR training OR Kinesiotherapy OR kinesiatrics OR "Cardiac Rehabilitation") AND ("Hypertrophic cardiomyopathy" OR "Hypertrophic Obstructive Cardiomyopathy" OR "Hypertrophic nonobstructive cardiomyopathy" OR "Hypertrophic non-obstructive cardiomyopathy" OR HCM OR HOCM) | All Field | 3,662 |
| Cochrane | (Exercise* OR "Physical Activit*" OR walking OR sport* OR training OR Kinesiotherapy OR kinesiatrics OR "Cardiac Rehabilitation") AND ("Hypertrophic cardiomyopathy" OR "Hypertrophic Obstructive Cardiomyopathy" OR "Hypertrophic nonobstructive cardiomyopathy" OR "Hypertrophic non-obstructive cardiomyopathy" OR HCM OR HOCM) | All Field | 258 |
| WOS | (Exercise* OR "Physical Activit*" OR walking OR sport* OR training OR Kinesiotherapy OR kinesiatrics OR "Cardiac Rehabilitation") AND ("Hypertrophic cardiomyopathy" OR "Hypertrophic Obstructive Cardiomyopathy" OR "Hypertrophic nonobstructive cardiomyopathy" OR "Hypertrophic non-obstructive cardiomyopathy" OR HCM OR HOCM) | All Field | 5,090 |
| SCOPUS | TITLE-ABS-KEY ( ( exercise* OR "Physical Activit*" OR walking OR sport* OR training OR kinesiotherapy OR kinesiatrics OR "Cardiac Rehabilitation" ) AND ( "Hypertrophic cardiomyopathy" OR "Hypertrophic Obstructive Cardiomyopathy" OR "Hypertrophic nonobstructive cardiomyopathy" OR "Hypertrophic non-obstructive cardiomyopathy" OR hcm OR hocm ) ) | Title, Abstract, Keywords | 4,546 |
| EMBASE | #3.  #1 AND #2                                                3,270  #2.  'hypertrophic cardiomyopathy':ti,ab,kw OR               34,314       'hypertrophic obstructive       cardiomyopathy':ti,ab,kw OR 'hypertrophic       nonobstructive cardiomyopathy':ti,ab,kw OR       hcm:ti,ab,kw OR hocm:ti,ab,kw  #1.  'exercise':ti,ab,kw OR 'physical                     1,571,437       activity':ti,ab,kw OR 'walking':ti,ab,kw OR       'sport':ti,ab,kw OR 'training':ti,ab,kw OR       'kinesiotherapy':ti,ab,kw OR       kinesiatrics:ti,ab,kw OR 'heart       rehabilitation':ti,ab,kw OR 'cardiac       rehabilitation':ti,ab,kw | All Field | 3,270 |
